# Development of thalamocortical connections between the mediodorsal thalamus and the prefrontal cortex and its implication in cognition

**DOI:** 10.3389/fnhum.2014.01027

**Published:** 2015-01-09

**Authors:** Brielle R. Ferguson, Wen-Jun Gao

**Affiliations:** Department of Neurobiology and Anatomy, Drexel University College of MedicinePhiladelphia, PA, USA

**Keywords:** mediodorsal nucleus of thalamus, prefrontal cortex, development, cognitive function, psychiatric disorders

## Abstract

The mediodorsal thalamus (MD) represents a fundamental subcortical relay to the prefrontal cortex (PFC), and is thought to be highly implicated in modulation of cognitive performance. Additionally, it undergoes highly conserved developmental stages, which, when dysregulated, can have detrimental consequences. Embryonically, the MD experiences a tremendous surge in neurogenesis and differentiation, and disruption of this process may underlie the pathology in certain neurodevelopmental disorders. However, during the postnatal period, a vast amount of cell loss in the MD occurs. These together may represent an extended critical period for postnatal development, in which disturbances in the normal growth or reduction of the MD afferents to the PFC, can result in PFC-dependent cognitive, affective, or psychotic abnormalities. In this review, we explore the current knowledge supporting this hypothesis of a protracted critical period, and propose how developmental changes in the MD contribute to successful prefrontal cortical development and function. Specifically, we elaborate on the unique properties of MD-PFC connections compared with other thalamocortical afferents in sensory cortices, examine how MD-PFC innervation modulates synaptic transmission in the local prefrontal circuitry, and speculate on what occurs during postnatal development, particularly within the early neonatal stage, as well as juvenile and adolescent periods. Finally, we discuss the questions that remain and propose future experiments in order to provide perspective and novel insights into the cause of neuropsychiatric disorders associated with MD-PFC development.

## Introduction

The prefrontal cortex (PFC) was originally defined as the projection area of the mediodorsal (MD) thalamus (Rose and Woolsey, 1948), and this was further confirmed with anatomical studies in rodent (Guldin et al., 1981; Groenewegen, 1988; Uylings et al., 2003) and primate brains (Goldman-Rakic and Porrino, 1985; Giguere and Goldman-Rakic, 1988). With its critical roles in cognitive processes, such as working memory, attention, and cognitive flexibility, illumination of the subcortical regulation of the PFC is fundamental to further understanding these abilities. More than a half-century since the initial observation of the MD’s extensive reciprocal innervation of the PFC, numerous studies have focused on understanding the role of the MD network in cognitive function (see reviews, Kuroda et al., 1998; Ongür and Price, 2000; Constantinidis and Procyk, 2004; Watanabe and Funahashi, 2012; Baxter, 2013; Funahashi, 2013; Mitchell and Chakraborty, 2013). However, the functional relevance of the MD-PFC circuitry has remained somewhat elusive, specifically how developmental changes in the MD contribute to prefrontal cortical development and function.

A recent study reported an excess of neurons in the human newborn MD compared with that of the adult, suggesting a potential role for extended developmental processes to regulate cell survival in this region (Abitz et al., 2007). In rodents, during the first postnatal week, MD afferent arrival precedes PFC lamination, and thus has been hypothesized to be instrumental to proper differentiation, synaptic organization, and circuit formation in the early neonatal stage (Van Eden, 1986). Yet, behavioral data suggest that MD input is most critical in late postnatal development, such as juvenile and adolescent periods, in which disturbance of MD activity can have deleterious consequences for the execution of PFC-dependent functions like working memory (Vicedomini et al., 1982). This raises the interesting question of whether there is a time window in which MD input is most influential for PFC development. Further, it calls for a more detailed exploration of how MD activity is able to shape the maturation of the PFC circuitry during postnatal development.

It is widely believed that cortical development is experience and activity dependent. Thus, with the MD providing the majority of excitatory innervation to the PFC, determining how MD activity and afferent fibers affect the development of executive functioning is of considerable importance. The advent of advanced experimental techniques that allow for precise dissection of whether and how a nodal dysfunction in a cognitive circuit affects the development of certain mental attributes represents an extremely exciting time for inquiry in this field. With this in mind, we will return to the seminal anatomical and lesion data that suggest an expanded window for the MD to regulate PFC maturation. Using this as a guide, we will suggest potential mechanisms through which the MD may affect PFC development. Then, after discussing briefly what is known about the MD’s involvement in cognition in adulthood, we will propose experiments and discuss future directions in the realm of understanding the MD thalamus and its role in the development of successful PFC-associated cognition.

## MD-PFC development

Using tract-tracing techniques, early studies indicate that in rodents, MD fibers have arrived in the PFC by birth. These fibers can be found densely coursing through the deeper cortical levels, which will develop prior to the superficial layers. However, what is most intriguing is the sizable projection present in the upper cortical plate, what will later become layer III (Van Eden, 1986). This is in contrast to the developmental trajectory in primary sensory cortices, where thalamocortical innervation occurs days later around postnatal day 4 (P4; Wise and Jones, 1978). The majority of MD innervation of the PFC takes place in layer III (Leonard, 1969; Krettek and Price, 1977). Given that MD projection arrival predates the development of its site of termination, it has been suggested that this innervation may shape the future dendritic architectures of layer III PFC neurons, more so than other thalamocortical afferents in primary sensory cortices (van Eden et al., 1990). The density of this MD projection will continue to increase until it peaks at P10, once cortical differentiation of layer III occurs. However, in the early juvenile stage, at P13, a profound reduction in innervation has been reported through retrograde tract-tracing experiments in mice (Rios and Villalobos, 2004). After P16, the mean average shows a slight increase until P60, after which afferent density remains relatively stable (Rios and Villalobos, 2004).

**Figure 1:**
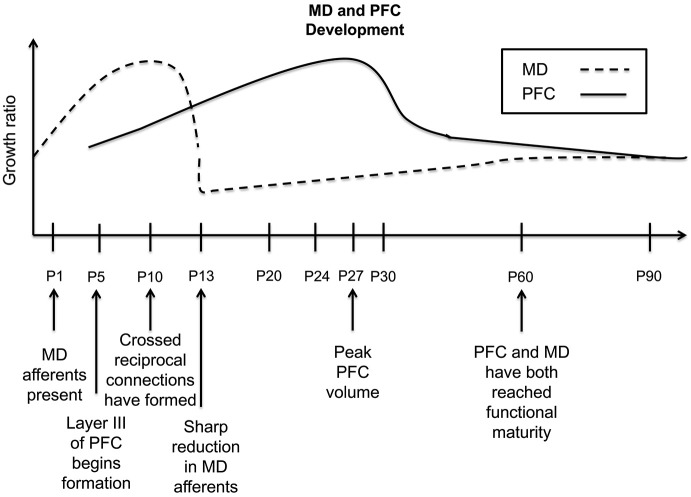
**Timeline of the developmental trajectory of MD afferent density and PFC volume in the rodent PFC**. Dashed black line—MD afferent density to the PFC, solid black line—PFC volume. MD afferent arrival precedes the differentiation and lamination of layer III; the density of this projection increases through P10, then decreases sharply at P13, and stabilizes by P60. PFC volume begins increasing at P5, reaching its peak around P27, dropping rapidly around P30, and then decreasing gradually until P90. Given that the PFC volumetric changes follow closely behind the density dynamics of inputs from the MD, this may suggest a regulatory relationship of MD innervation on PFC development. This summary graph is based on the previous publications (Van Eden and Uylings, 1985; Van Eden, 1986; Rios and Villalobos, 2004).

Notably, changes in the quantity of MD fibers appear to mirror and precede volumetric alterations in the PFC, further suggesting that the MD plays a critical regulatory role over prefrontal cortical development (Van Eden and Uylings, 1985; Van Eden, 1986; Rios and Villalobos, 2004). Following just days behind the arrival and subsequent increase in the density of MD afferent terminals, the PFC undergoes a vast increase in volume (see Figure 1), which peaks on average at P24 and P30, for medial and orbital PFC, respectively. Then a decrease occurs in juvenile PFC volume that follows an earlier decrease in the density of MD thalamocortical innervation (Van Eden and Uylings, 1985; Rios and Villalobos, 2004). Data suggest that this reduction in volume may be a reflection of a loss of dendritic complexity and spine density in PFC pyramidal neurons (Marmolejo et al., 2012). This could stem from a refinement of the proper synaptic contacts between the MD and PFC and axonal pruning in the PFC local circuitry, resulting in a net reduction in PFC volume. However, in humans, a profound reduction in MD neuronal number is observed over development. Specifically, the total quantity of neurons in the entire MD was an average of 41% lower in the adult vs. the newborn brain (Abitz et al., 2007). This is the first demonstration of a higher amount of neurons in the neonatal MD compared with the adult, although whether this is also the case for the rodent or primate brain is unclear. Nonetheless, the development of PFC circuitry seemingly under regulation of the MD, proceeds through specific developmental stages or critical windows. This is an intriguing possibility, but requires further experimentation in support of this claim. For example, whether this pattern is seen similarly across species, as well as demonstration of whether experimental reductions in MD afferent density can disrupt these PFC fluctuations would need to be explored. Whether PFC development is dictated through reductions in MD cell density, afferent innervation, or both, the ultimate mature phenotype of either structure is not attained until early adulthood.

Early evidence from animal studies suggests that primary sensory and motor cortices reach developmental maturity prior to regions of association or higher-order cortex such as the PFC (Guillery, 2005). Utilizing measures such as regional blood flow and glucose metabolism, researchers have demonstrated that similarly in humans, the frontal regions show a delayed maturation in comparison with sensorimotor areas (Chiron et al., 1992; Chugani, 1998). Thalamocortical synaptic connectivity in the PFC continues to increase into the late teens (Alkonyi et al., 2011), while myelination, a marker of mature axons, has been suggested to not be complete until the fourth decade of life (Yaklovev and Lecours, 1967). While all regions seem to proceed through distinct phases, including an overproduction of spines and a subsequent reduction, this process is elongated in the PFC. Additionally, the magnitude of this overproduction is exaggerated in the PFC with a tremendous excess in spine number, as well as a slower rate of pruning (Elston et al., 2009). This significant delay is critical because it creates a protracted window for neuronal activity from other brain regions, such as the MD, to mold the development of the PFC (Kolb et al., 2012). It may even allow for greater levels of metaplasticity (a form of activity-dependent changes in neural functions that modulate subsequent synaptic plasticity) than regions with shorter developmental windows (e.g., primary sensory cortices). Through allowing for such a high level of overproduction, there is an enormous potential for experience and environmental influences to affect how the PFC will mature. However, this creates a double-edged sword, given that the PFC needs this plasticity to allow for maximal development of higher order cognitive functions, but it also leaves this brain region in a delicate position to be more easily compromised by various insults, such as stress. This highlights an important window from birth to early adulthood, wherein developmental insults may dysregulate the proper pattern of reciprocal innervation between the MD and PFC.

**Figure 2:**
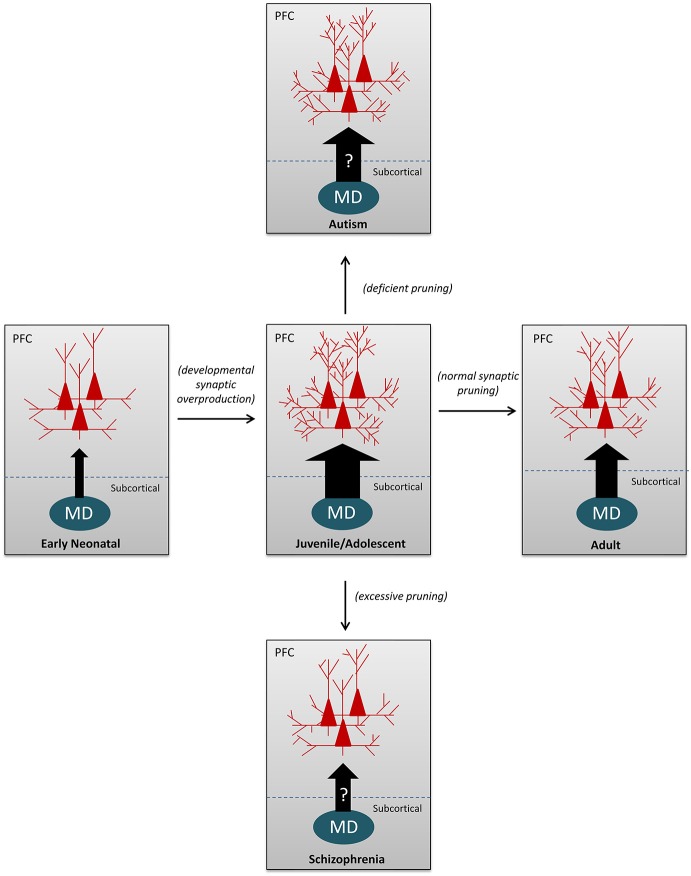
**Schematic illustrations to depict the MD-PFC afferents and synaptic pruning under both normal and abnormal developmental conditions**. The arrow sizes represent the relative numbers/densities of MD-PFC afferent fibers; whereas the number of dendritic branches in the PFC pyramidal neurons reflect changes in synaptic density and dendritic complexity. During normal development, both thalamocortical afferent fibers and dendritic branches of pyramidal neurons in the PFC are underdeveloped in the early neonate, over-produced during the juvenile and adolescent periods, and then reduced to normal levels in adulthood by eliminating the excess presynaptic axonal arbors (thalamocortical fibers) and/or postsynaptic dendrites. In contrast, as speculated, decreases in MD activity could result in a loss of synaptic drive to the PFC early in development, leading to a decrease in synaptic density, as is observed in patients with schizophrenia. Conversely, a presumably overactive thalamocortical drive to the PFC, could lead to a failure of the normal developmental synaptic pruning, resulting in increased spine density or hyperconnectivity, as demonstrated in autism.

Accordingly, disturbances localized at both nodes along this pathway have been demonstrated to be present in different psychiatric disorders of potential developmental origin, such as schizophrenia, autism, and depression. Yet, how the MD-PFC connections are affected in the disease state remains to be determined. For example, MD thalamic volume or cell number (Pakkenberg, 1990; Popken et al., 2000; Young et al., 2000) and activity dysregulation (Andrews et al., 2006; Minzenberg et al., 2009) have been implicated in schizophrenia neuropathology. Thus, it has been speculated that decreases in MD activity could result in a loss of synaptic drive to the PFC early in development, leading to a decrease in PFC synaptic density (Minzenberg and Carter, 2012). This hypoinnervation may also underlie the gray matter reductions that have been observed in schizophrenic patients in adulthood (Zipursky et al., 1992; Schlaepfer et al., 1994). Conversely, the reverse situation could be hypothesized, where there is an overactive thalamocortical drive to the PFC, leading to a failure of normal developmental synaptic pruning, as has been demonstrated recently to take place in autism (Tang et al., 2014), i.e., a hyperconnectivity in the PFC (Belmonte et al., 2004). Concurrently, research points to a correlation between thalamus and total brain volume that is preserved in healthy controls and children with autism (Hardan et al., 2008), but lost in samples that incorporate adults with the disease (Tsatsanis et al., 2003; Hardan et al., 2006). However, interpretation of these findings becomes difficult in light of imaging data demonstrating a hypoactivation of the thalamus that occurs in autism spectrum disorders (Buchsbaum et al., 1992; Baron-Cohen et al., 1999). Although the nature of the thalamic disruption in autism is unclear, there seems to be a dysregulation of the normal reciprocal synaptic relationship between the thalamus in the cortex that occurs during development (Fair et al., 2010; Righi et al., 2014). If MD afferent quantity is strictly coordinating PFC synaptic density, hypothetically, thalamic abnormalities could disrupt the normal developmental reduction in synapse number resulting in an over-pruning or under-pruning and compromising PFC function (see Figure 2). Thus, it is critical to illuminate what specific alterations in MD-PFC connections occur in the disease condition to better elucidate how these changes contribute to PFC-dependent behavioral deficits.

## MD-PFC connectivity

The rodent MD is delineated into four major regions, central, medial, lateral, and paralamellar (Krettek and Price, 1977), but can be more broadly grouped into the central/medial and lateral regions (Van Eden, 1986). Similarly, in primates, there is a medial magnocellular portion (MDmc), a lateral parvocellular subdivision (MDpc), but also a lateral multiformis and densocellular portion (Goldman-Rakic and Porrino, 1985; Giguere and Goldman-Rakic, 1988; Barbas et al., 1991). The MDmc targets areas more medially situated in the PFC, such as the ventral and medial PFC, while the MDpc innervates the dorsolateral and dorsomedial areas (Giguere and Goldman-Rakic, 1988; Barbas et al., 1991). As mentioned, these afferents innervate primarily layer III, but also sparsely layer I (in rodents) and layer IV (in primates), while reciprocal connections originate from layer VI and V (Krettek and Price, 1977; Van Eden, 1986; Giguere and Goldman-Rakic, 1988; Groenewegen, 1988). Interestingly, in rodents, the destinations of these different MD areas show distinct developmental patterns, further strengthening the possibility of MD activity regulating the maturation of its target region. The medial MD innervates the prelimbic, dorsal and ventral agranular insular cortex, and these regions show a peak in volumetric density six days prior to the dorsal anterior cingulate cortex (dACC) and medial precentral cortex, areas innervated by the lateral MD (Van Eden, 1986). PFC axons intended for the MD form reciprocal connections between P4 and P10, prior to the peak and later decrease in PFC volume (Van Eden, 1986). This reciprocal innervation is mediated by glutamatergic neurotransmission between the two regions (Pirot et al., 1994; Sherman, 2014).

MD projections synapse directly onto apical dendrites of pyramidal neurons in the PFC (Kuroda et al., 1995; Négyessy et al., 1998). This would imply that coincident with functional synaptic activity, there is the capacity for glutamate release to cause α-Amino-3-hydroxy-5-methyl-4-isoxazolepropionic acid (AMPA) and N-methyl-D-aspartate (NMDA) facilitated depolarization of PFC neurons. Given that cortical development is believed to be activity-dependent (Katz and Shatz, 1996; Catalano et al., 1997), it is likely that the MD’s glutamatergic innervation has functional consequences for the maturation of PFC neurotransmission, and this will be elaborated upon in the following sections. Through neuronal processes such as activity-dependent upregulation of the expression of certain neurotrophins like brain derived neurotrophic factor (BDNF; Lu, 2003), as well as NMDA-mediated facilitation of calcium influx and subsequent intracellular signaling cascades (Marmolejo et al., 2012), the MD is in a position to exert a critical regulatory role over plasticity and maturation in the PFC.

## Effects of MD-PFC afferents on excitatory neurotransmission in the PFC

Synapse development, fine-tuning, and maintenance are heavily reliant on early developmental activity (Goodman and Shatz, 1993). NMDA receptor (NMDAR) function in the PFC, which is pivotal in these processes, is widely recognized as operating importantly in cognitive abilities (Wang et al., 2008, 2013), as well as being linked to cognitive dysfunction in schizophrenia (Snyder and Gao, 2013). During development, NMDARs undergo a developmental shift where the decay time of NMDAR-evoked excitatory currents shortens (Sheng et al., 1994; Flint et al., 1997). Underlying this decrease in NMDAR current decay time is a shift in subunit expression, particularly a switch between the NR2B and NR2A subunit (Monyer et al., 1994). Further, this shift is activity-dependent and is correlated with associative learning. Thus, disruption of early activity prevents this normal maturation (Carmignoto and Vicini, 1992) and learning capability (Dumas, 2005). Given this evidence, it is possible that MD glutamatergic innervation of the PFC helps to facilitate the activation and maturation of NMDARs in the PFC, and thus is critical in plasticity and synapse refinement underlying learning and memory processes.

Data from sensory cortices suggest that a loss of excitatory input would impair the developmental progression of excitatory synapse refinement (Catalano et al., 1997; Quinlan et al., 1999). However, NMDAR development in the PFC appears to be unique and specialized, differing from other cortical areas. Specifically, the PFC lacks a significant NR2B to NR2A subunit switch during development, with the adult level of NR2B-containing NMDARs significantly higher than that of the primary visual cortex (Wang et al., 2008). Thus, the implications for MD disruption on excitatory neurotransmission in the PFC are likely complex and distinct from what occurs in other cortical areas. The effects of a lesion or reduced activity in the MD on PFC NMDAR maturation have yet to be studied, and this certainly represents a vital inquiry to be explored. At present, it remains unclear how MD-PFC afferents shape PFC circuit formation and maintain the excitatory/inhibitory (E/I) balance during development.

## Effects of MD-PFC afferents on inhibitory neurotransmission in the PFC

Gamma-aminobutyric acid (GABA)-ergic neurotransmission follows a developmental trajectory in which GABA release from immature neurons onto a post-synaptic cell results in an excitatory depolarization of neurons during the first postnatal week (Ben-Ari et al., 1989; Staley et al., 1995; Owens et al., 1996; Dammerman et al., 2000). This excitation is a product of the distribution of the chloride (Cl-) pumps, Na-K-Cl cotransporter (NKCC1) and potassium-chloride transporter member 5 (aka: KCC2), that pump negatively charged Cl- into and out of the cell respectively. Early in development there is an upregulation of the expression of KCC2, causing a substantial increase in Cl- extrusion from the cell (Lu et al., 1999; Rivera et al., 1999). Thus, when GABA binds GABA_A_ receptors, Cl- rushes into the cell, causing hyperpolarization. It has been suggested that the fast inhibitory synaptic neurotransmission facilitated by the release of GABA, specifically from fast-spiking inhibitory interneurons, may help generate oscillations in groups of neurons that underlie certain types of cognitive performance, such as working memory and attention (Gonzalez-Burgos and Lewis, 2008). Indeed, that would make proper regulation of this shift of GABA’s reversal potential (E_GABA_) in the PFC from a depolarized to a hyperpolarized potential, critical for proper development of cognitive abilities.

A crucial role for GABA neurotransmission in facilitating neurodevelopment has been emerging in the developmental literature (Barker et al., 1998; Owens and Kriegstein, 2002). Functional GABA_A_ receptors are present embryonically (Métin et al., 2000) and it has been demonstrated that these receptors have a higher affinity for GABA and are less vulnerable to early desensitization relative to their adult counterparts (Owens et al., 1999). Further, in the developing cortex, GABAergic synapses constitute almost half of all synaptic junctions (De Felipe et al., 1997). This allows for spontaneous GABA release perinatally to have profound physiological effects on neurons expressing GABA receptors, and also to act as a potent signaling molecule in neuronal development (Wolff et al., 1978). Moreover, it suggests that afferent drive of GABAergic neurons in the PFC may play a significant role in shaping the prolonged development of the PFC. Yet, this assumption remains to be directly examined.

MD projections target both glutamatergic pyramidal neurons and GABAergic interneurons in the PFC (Kuroda et al., 2004; Negyessy and Goldman-Rakic, 2005; Rotaru et al., 2005). This has implications when considering facilitation of GABAergic activity in the mature PFC, but also has important consequences for investigating the neurodevelopment of prefrontal inhibitory circuitry. Data suggests that GABA release in the early developing cortex is able to facilitate the transition of cellular responses to GABA from depolarizing to hyperpolarizing (Ganguly et al., 2001). Although this is mainly hypothesized to be an action potential-independent process (Ganguly et al., 2001), depolarization of GABAergic interneurons by thalamocortical afferents may increase the likelihood of this spontaneous release. Although controversial (Leinekugel et al., 1995; Ben-Ari et al., 1997), AMPA receptor-mediated excitation has been shown to play a role in the facilitation of GABA-induced depolarization. Thus, the MD could provide a portion of the excitatory drive to GABA interneurons, helping developmentally regulate the maturation of GABAergic neurotransmission in the PFC. Coincidentally, the increase in spontaneous GABAergic currents corresponds with the significant increase in MD innervation of the PFC, and peaks when this afferent input is at its greatest height (Van Eden, 1986; Ben-Ari et al., 1989). This is purely speculative, however, and further experimentation, including repetition of these experiments in PFC neurons *in vivo* and *ex vivo* throughout development, would be necessary to support this hypothesis. If the MD is indeed facilitating this GABA shift, this would have profound consequences for functional connectivity in the PFC, and cognitive abilities that rely heavily on GABA-driven network activity, as well as the E/I balance in the PFC circuitry.

## Lesions and their implications for MD-PFC development

The MD has been implicated in a number of neuropathological diseases, such as schizophrenia, autism, and depression. When you consider MD disruption in the context of the late maturity of the PFC, it has fundamental ontogenic implications. To explore these questions, a number of researchers have employed an array of lesion methods at various points in development and have studied the effects on PFC-dependent functions.

Strengthening the claim that the MD regulates PFC function in adulthood is the plethora of data indicating that lesions of the MD result in deficits in PFC-dependent abilities. For example, MD lesions impair performance in various tasks that measure working memory and executive function, such as the T-maze, radial maze, and set-shifting (Winocur, 1985; Stokes and Best, 1988; Hunt and Aggleton, 1991; Block et al., 2007). However, for many of these cognitive tasks, there are negative data suggesting just the opposite: the MD functions minimally in the execution of these abilities (Beracochea et al., 1989; Neave et al., 1993). These contradictory results could partially be attributed to the variety of the methods used for employing these lesions in early studies. However, the development of more sophisticated lesioning methodology has painted a clearer picture of MD function in the recent decades.

Data regarding subregion specificity of deficits is less robust in rodents, given that the majority of studies employ bilateral lesions of the structure in its entirety. However, in primates the relatively well-delineated boundaries of the MD have allowed for localized lesions and demonstration of specific patterns of deficits. For example, excitotoxic and neurotoxic lesions of the MDmc result in deficits in reinforcer devaluation and scene learning, skills that depend on an intact orbital frontal cortex, the recipient of MDmc fibers (Mitchell et al., 2007a,b). The dlPFC is implicated heavily in working memory processes, and accordingly, lesions involving the lateral MD cause impairments in spatial delayed alternation and delay-response tasks. Additionally, lateral MD neurons seem to represent cue, delay, and response related information necessary for performing a delayed-response task (Tanibuchi and Goldman-Rakic, 2003). Data such as this led to the dogma that the MD is simply relaying information from its extensive afferent network for use by the PFC for execution of cognitive tasks.

Obfuscating this simple congruity of major MD sub-region to its primary cortical target, however, is data indicating that medial MD lesions also cause deficits in delayed non-match to sample tasks, a measure of working memory. In addition, deficits seen following lesions seem to apply more heavily to the integration of newly learned rules or information necessary for completion of tasks, and not re-application of previously learned rules or strategies, which are affected by compromising the function of the corresponding MD projection regions. For example, MDm lesions do not impair retention of pre-operatively learned scenes in a scene learning task, but animals show significant deficits when learning novel scenes (Mitchell et al., 2008; Mitchell and Gaffan, 2008). A similar pattern may exist with strategy implementation as well (Mitchell et al., 2007a). Parallels are also seen in humans with diencephalic damage; patients with lesions which are restricted to the thalamus show fewer instances of retrograde amnesia (Baxter, 2013). This suggests, that the MD generally may play a stronger role in integration and processing of novel information for use by the PFC, challenging the long-standing hypothesis of its function as a passive relay. This topic has been discussed thoroughly in many recent excellent reviews (Baxter, 2013; Funahashi, 2013; Mitchell and Chakraborty, 2013).

An emerging thread in early research, that has been left somewhat dormant in the recent years is exploring differential outcomes of lesions of both the PFC and MD at varying developmental timepoints. Conclusions from this pioneering work suggest that age at time of lesion is critical, and has important implications for cortical reorganization as well as functional recovery (Kolb and Gibb, 2007). Based on the timeline of MD-PFC development, it is not difficult to hypothesize that morphological changes and the extent of functional alterations would be an age-dependent phenomenon.

### Effects of lesions of the PFC across development on MD-PFC connections

Early studies suggested that frontal lesions in adult rodents resulted in substantial cognitive impairment, while animals with damage inflicted in early postnatal development had a much greater propensity for functional recovery (Kolb and Nonneman, 1978; Nonneman and Corwin, 1981). Correspondingly in primates, juvenile and adolescent lesions, specifically of the dlPFC, result in a profound impairment in delayed alternation performance (Goldman and Galkin, 1978), a behavior which is spared in prenatally lesioned animals. However, in an elegant series of experiments carried out by Kolb and colleagues, researchers demonstrated that there is a critical period that occurs during the second postnatal week in rats, within which the brain has a tremendous capability for recovery of connectivity and function following lesions. In contrast, prior to and following this time window, animals show profound impairment of PFC dependent abilities (Kolb and Gibb, 1990; Kolb et al., 1996, 1998). In concert, it was demonstrated following frontal lesions during this critical period in both rats and primates, coincident with functional recovery, there was a lack of retrograde degeneration observed in the MD (Goldman and Galkin, 1978; Kolb and Nonneman, 1978). In fact, van Eden et al. reported that in neonatally lesioned animals, MD neuronal cell density was significantly increased. However, after lesions in adults, when animals present with cognitive impairments, a significant degeneration of MD thalamic neurons has been observed (Kolb et al., 1974; Goldman and Galkin, 1978). This finding led to the hypothesis that the MD may be underlying sparing of PFC-dependent function observed in younger animals (Kolb and Nonneman, 1978). However, others were unable to demonstrate a reorganization of thalamocortical fibers following neonatal prefrontal lesions (de Brabander et al., 1991; Kolb et al., 1994).

Still, this does not rule out the ability of the MD to regulate PFC development and facilitate functional recovery following PFC lesions. The lack of MD neuronal loss following frontal damage that occurs in younger animals, the period in which functional recovery is the most substantial, lends credence to this claim (Goldman and Galkin, 1978; Kolb and Nonneman, 1978; Nonneman and Corwin, 1981). A failure to reorganize projections does not imply that the remaining MD projections do not play a role in sustaining cognitive function. Accordingly, an increase in dendritic complexity in other projection areas has been observed, which may function in rendering PFC-lesioned animals indistinguishable from controls in cognitive tasks (Kolb et al., 1994).

### Effects of lesions of the MD across development on MD-PFC connections

Unlike the PFC, early neonatal lesions (P1) of the MD produce negligible deficits in working memory (van Eden et al., 1994). The timing of this lesion prior to the substantial increase in the density of this projection (Van Eden, 1986) may allow for an ample compensation from other thalamic inputs, but this is purely speculative. Similar to PFC lesions occurring in the second postnatal week, however, MD disruption at P8–P10 also results in normal performance in spatial alternation behavior (Vicedomini et al., 1982). Interestingly though, despite the lack of working memory deficits in adulthood, early developmental disruption of the MD at P4 leads to a significant reduction in both the amount of dendrites and dendritic spines in the PFC (Marmolejo et al., 2012). This finding suggests that there may be subtler alterations in PFC synaptic connectivity, which may manifest itself in impairments in more cognitively taxing PFC-dependent tasks such as attentional set-shifting or the five-choice serial reaction task. Nonetheless, interruption of MD function at later points, from the juvenile stage onward results in profound deficits in spatial alternation behavior, and a wealth of other behavioral tasks, suggesting the later stages of PFC maturation rely heavily on the MD’s excitatory innervation (Vicedomini et al., 1982).

## Future directions and insights

The rise of the use of cutting edge techniques represents an exciting point in the exploration of the role the MD plays in PFC development. With the advent of optogenetics allowing for temporal control of cellular populations with millisecond precision (Boyden et al., 2005), this allows researchers to answer important questions about how the MD is functioning in certain PFC-dependent abilities in an acute manner. For example, this may endow researchers to answer with more certainty the depth of the MD’s role in regulating important cognitive functions in adulthood, for which the data thus far has not been entirely consistent. A particular interest is the use of Designer Receptors Exclusively Activated by Designer Drugs (DREADDs; Armbruster et al., 2007; Alexander et al., 2009) for probing PFC circuit function. This novel technology allows for pharmacogenetic downregulation of specific populations of cells, such as those in the MD. It utilizes ligand-driven activation of receptors, with a drug that can exert its pharmacological action for hours, without the need of continuous light or electrical stimulation. This provides an advantage when exploring the effects of subchronic inhibition in distinct developmental periods. Interestingly, a recent elegant study reported that inhibition of the MD with DREADDs in adult rats can disrupt thalamocortical connectivity and PFC-dependent cognition (Parnaudeau et al., 2013). Following this approach, researchers can begin to explore the major questions raised by this review. Primarily, is MD activity necessary for normal PFC development in the early neonatal period, as anatomical data might suggest? Outputs could include behavior, electrophysiology, as well as biochemical assays. How does MD inhibition in early development affect executive functioning in adulthood? Researchers could also explore the consequences of MD inhibition for single cell activity in the PFC using whole-cell recordings, or groups of neurons while performing cognitive tasks using *in vivo* recordings. This would allow for the precise correlation of neuronal firing patterns with varying levels of cognitive performance, potentially highlighting targets for rescue of cognitive ability. At a more global level, oscillations and synchrony could be monitored to answer whether early MD dysregulation results in changes in frontal synchrony coincident with behavioral impairment in adulthood. Finally, with each of these avenues is an opportunity for the systematic exploration of the underlying pathological mechanisms associated with these changes in function and physiological activity. Thus, an investigation of changes in certain enzymes and receptor distribution, such as those related to excitatory and inhibitory synaptic transmission and plasticity using biochemical assays would be warranted as well. It is critical to explore how MD inhibition affects the maturation of particular receptors and their subtype components in the PFC local circuitry, such as AMPARs, NMDARs or GABARs, all of which are linked to successful PFC-dependent cognitive function. Through this exploration, we can provide important insights into the role of the MD in development of the PFC and related cognitive abilities, and provide novel approaches and targets for the treatment of neurodevelopmental disorders with which MD dysfunction is associated.

In summary, the MD represents a fundamental subcortical relay to the PFC, and is highly implicated in modulation of PFC-dependent cognitive and executive abilities. Further, more recent research challenges the dogma of its role as a passive relay, suggesting it has an important function in the integration of newly learned information for use by the PFC. If this is correct, this underscores the importance of this brain region as a cognitive center, lessening the likelihood of early MD dysregulation being without consequence for PFC maturation. As mentioned, we hypothesize that MD afferent activity may be important in regulation of PFC development earlier than previous data has indicated. Afferent fibers are present prior to thalamocortical afferents in sensory cortices while reductions in cell number and afferent density have been reported to occur into the juvenile stage and adolescence (Van Eden, 1986; Abitz et al., 2007). This may represent an extended critical period for postnatal development, in which disturbances in the normal growth or decrease of MD afferents can result in PFC-dependent cognitive, affective, or psychotic abnormalities. Therefore, it is imperative for us to further explore how developmental changes in the MD contribute to successful prefrontal cortical development and function. Specifically, it is important to better elucidate the unique properties of MD-PFC connections compared with other thalamocortical afferents in sensory cortices, and to examine how MD-PFC innervation affects synaptic transmission and E/I balance in the local prefrontal circuitry. Finally, we must explore the functional importance of MD-PFC connections in psychiatric disorders such as autism, depression, attention deficit hyperactivity disorder, and schizophrenia. It is our belief that answering these questions will not only enhance our understanding of the MD and PFC function in normal cognition, but also provide perspectives into the cause and pathophysiological processes of neuropsychiatric disorders linked with MD-PFC development.

## Conflict of interest statement

The authors declare that the research was conducted in the absence of any commercial or financial relationships that could be construed as a potential conflict of interest.

## Abbreviations

AMPA: α-amino-3-hydroxy-5-methyl-4-isoxazolepropionic acid
GABA: gamma-aminobutyric acid
MD: mediodorsal nucleus of thalamus
NMDA: N-methyl-D-aspartate
PFC: prefrontal cortex.

## References

[B1] Abitz M. Nielsen R. D. Jones E. G. Laursen H. Graem N. Pakkenberg B. (2007). Excess of neurons in the human newborn mediodorsal thalamus compared with that of the adult. Cereb. Cortex 17, 2573–2578. 10.1093/cercor/bhl163 17218480

[B2] Alexander G. M. Rogan S. C. Abbas A. I. Armbruster B. N. Pei Y. Allen J. A.. (2009). Remote control of neuronal activity in transgenic mice expressing evolved G protein-coupled receptors. Neuron 63, 27–39. 10.1016/j.neuron.2009.06.014 19607790 PMC2751885

[B3] Alkonyi B. Juhász C. Muzik O. Behen M. E. Jeong J.-W. Chugani H. T. (2011). Thalamocortical connectivity in healthy children: asymmetries and robust developmental changes between ages 8 and 17 years. AJNR Am. J. Neuroradiol. 32, 962–969. 10.3174/ajnr.a2417 21454411 PMC3095749

[B4] Andrews J. Wang L. Csernansky J. G. Gado M. H. Barch D. M. (2006). Abnormalities of thalamic activation and cognition in schizophrenia. Am. J. Psychiatry 163, 463–469. 10.1176/appi.ajp.163.3.463 16513868

[B5] Armbruster B. N. Li X. Pausch M. H. Herlitze S. Roth B. L. (2007). Evolving the lock to fit the key to create a family of G protein-coupled receptors potently activated by an inert ligand. Proc. Natl. Acad. Sci. U S A 104, 5163–5168. 10.1073/pnas.0700293104 17360345 PMC1829280

[B6] Barbas H. Henion T. H. Dermon C. R. (1991). Diverse thalamic projections to the prefrontal cortex in the rhesus monkey. J. Comp. Neurol. 313, 65–94. 10.1002/cne.903130106 1761756

[B7] Barker J. L. Behar T. Li Y. X. Liu Q. Y. Ma W. Maric D.. (1998). GABAergic cells and signals in CNS development. Perspect. Dev. Neurobiol. 5, 305–322. 9777645

[B8] Baron-Cohen S. Ring H. A. Wheelwright S. Bullmore E. T. Brammer M. J. Simmons A.. (1999). Social intelligence in the normal and autistic brain: an fMRI study. Eur. J. Neurosci. 11, 1891–1898. 10.1046/j.1460-9568.1999.00621.x 10336657

[B9] Baxter M. G. (2013). Mediodorsal thalamus and cognition in non-human primates. Front. Syst. Neurosci. 7:38. 10.3389/fnsys.2013.00038 23964206 PMC3734369

[B11] Belmonte M. K. Allen G. Beckel-Mitchener A. Boulanger L. M. Carper R. A. Webb S. J. (2004). Autism and abnormal development of brain connectivity. J. Neurosci. 24, 9228–9231. 10.1523/jneurosci.3340-04.2004 15496656 PMC6730085

[B12] Ben-Ari Y. Cherubini E. Corradetti R. Gaiarsa J. L. (1989). Giant synaptic potentials in immature rat CA3 hippocampal neurones. J. Physiol. 416, 303–325. 2575165 10.1113/jphysiol.1989.sp017762 PMC1189216

[B13] Ben-Ari Y. Khazipov R. Leinekugel X. Caillard O. Gaiarsa J. L. (1997). GABAA, NMDA and AMPA receptors: a developmentally regulated ‘ménage à trois’. Trends Neurosci. 20, 523–529. 10.1016/s0166-2236(97)01147-8 9364667

[B14] Beracochea D. J. Jaffard R. Jarrard L. E. (1989). Effects of anterior or dorsomedial thalamic ibotenic lesions on learning and memory in rats. Behav. Neural Biol. 51, 364–376. 10.1016/s0163-1047(89)91000-5 2730499

[B15] Block A. E. Dhanji H. Thompson-Tardif S. F. Floresco S. B. (2007). Thalamic-prefrontal cortical-ventral striatal circuitry mediates dissociable components of strategy set shifting. Cereb. Cortex 17, 1625–1636. 10.1093/cercor/bhl073 16963518

[B16] Boyden E. S. Zhang F. Bamberg E. Nagel G. Deisseroth K. (2005). Millisecond-timescale, genetically targeted optical control of neural activity. Nat. Neurosci. 8, 1263–1268. 10.1038/nn1525 16116447

[B17] Buchsbaum M. S. Siegel B. V. Jr. Wu J. C. Hazlett E. Sicotte N. Haier R.. (1992). Brief report: attention performance in autism and regional brain metabolic rate assessed by positron emission tomography. J. Autism Dev. Disord. 22, 115–125. 10.1007/bf01046407 1592761

[B18] Carmignoto G. Vicini S. (1992). Activity-dependent decrease in NMDA receptor responses during development of the visual cortex. Science 258, 1007–1011. 10.1126/science.1279803 1279803

[B19] Catalano S. M. Chang C. K. Shatz C. J. (1997). Activity-dependent regulation of NMDAR1 immunoreactivity in the developing visual cortex. J. Neurosci. 17, 8376–8390. 9334411 10.1523/JNEUROSCI.17-21-08376.1997 PMC6573766

[B20] Chiron C. Raynaud C. Mazière B. Zilbovicius M. Laflamme L. Masure M. C.. (1992). Changes in regional cerebral blood flow during brain maturation in children and adolescents. J. Nucl. Med. 33, 696–703. 1569478

[B21] Chugani H. T. (1998). A critical period of brain development: studies of cerebral glucose utilization with PET. Prev. Med. 27, 184–188. 10.1006/pmed.1998.0274 9578992

[B22] Constantinidis C. Procyk E. (2004). The primate working memory networks. Cogn. Affect. Behav. Neurosci. 4, 444–465. 10.3758/cabn.4.4.444 15849890 PMC3885185

[B23] Dammerman R. S. Flint A. C. Noctor S. Kriegstein A. R. (2000). An excitatory GABAergic plexus in developing neocortical layer 1. J. Neurophysiol. 84, 428–434. 10899216 10.1152/jn.2000.84.1.428

[B24] de Brabander J. M. van Eden C. G. de Bruin J. P. (1991). Neuroanatomical correlates of sparing of function after neonatal medial prefrontal cortex lesions in rats. Brain Res. 568, 24–34. 10.1016/0006-8993(91)91375-b 1814571

[B25] De Felipe J. Marco P. Fairén A. Jones E. G. (1997). Inhibitory synaptogenesis in mouse somatosensory cortex. Cereb. Cortex 7, 619–634. 10.1093/cercor/7.7.619 9373018

[B26] Dumas T. C. (2005). Developmental regulation of cognitive abilities: modified composition of a molecular switch turns on associative learning. Prog. Neurobiol. 76, 189–211. 10.1016/j.pneurobio.2005.08.002 16181726

[B27] Elston G. N. Oga T. Fujita I. (2009). Spinogenesis and pruning scales across functional hierarchies. J. Neurosci. 29, 3271–3275. 10.1523/jneurosci.5216-08.2009 19279264 PMC6666449

[B28] Fair D. A. Bathula D. Mills K. L. Dias T. G. Blythe M. S. Zhang D.. (2010). Maturing thalamocortical functional connectivity across development. Front. Syst. Neurosci. 4:10. 10.3389/fnsys.2010.00010 20514143 PMC2876871

[B29] Flint A. C. Maisch U. S. Weishaupt J. H. Kriegstein A. R. Monyer H. (1997). NR2A subunit expression shortens NMDA receptor synaptic currents in developing neocortex. J. Neurosci. 17, 2469–2476. 9065507 10.1523/JNEUROSCI.17-07-02469.1997 PMC6573498

[B30] Funahashi S. (2013). Thalamic mediodorsal nucleus and its participation in spatial working memory processes: comparison with the prefrontal cortex. Front. Syst. Neurosci. 7:36. 10.3389/fnsys.2013.00036 23914160 PMC3728470

[B31] Ganguly K. Schinder A. F. Wong S. T. Poo M. (2001). GABA itself promotes the developmental switch of neuronal GABAergic responses from excitation to inhibition. Cell 105, 521–532. 10.1016/s0092-8674(01)00341-5 11371348

[B32] Giguere M. Goldman-Rakic P. S. (1988). Mediodorsal nucleus: areal, laminar and tangential distribution of afferents and efferents in the frontal lobe of rhesus monkeys. J. Comp. Neurol. 277, 195–213. 10.1002/cne.902770204 2466057

[B33] Goldman P. S. Galkin T. W. (1978). Prenatal removal of frontal association cortex in the fetal rhesus monkey: anatomical and functional consequences in postnatal life. Brain Res. 152, 451–485. 10.1016/0006-8993(78)91103-4 99206

[B34] Goldman-Rakic P. S. Porrino L. J. (1985). The primate mediodorsal (MD) nucleus and its projection to the frontal lobe. J. Comp. Neurol. 242, 535–560. 10.1002/cne.902420406 2418080

[B35] Gonzalez-Burgos G. Lewis D. A. (2008). GABA neurons and the mechanisms of network oscillations: implications for understanding cortical dysfunction in schizophrenia. Schizophr. Bull. 34, 944–961. 10.1093/schbul/sbn070 18586694 PMC2518635

[B36] Goodman C. S. Shatz C. J. (1993). Developmental mechanisms that generate precise patterns of neuronal connectivity. Cell 72(Suppl.), 77–98. 10.1016/s0092-8674(05)80030-3 8428376

[B37] Groenewegen H. J. (1988). Organization of the afferent connections of the mediodorsal thalamic nucleus in the rat, related to the mediodorsal-prefrontal topography. Neuroscience 24, 379–431. 10.1016/0306-4522(88)90339-9 2452377

[B38] Guillery R. W. (2005). Is postnatal neocortical maturation hierarchical? Trends Neurosci. 28, 512–517. 10.1016/j.tins.2005.08.006 16126285

[B39] Guldin W. O. Pritzel M. Markowitsch H. J. (1981). Prefrontal cortex of the mouse defined as cortical projection area of the thalamic mediodorsal nucleus. Brain Behav. Evol. 19, 93–107. 10.1159/000121636 7326577

[B40] Hardan A. Y. Girgis R. R. Adams J. Gilbert A. R. Keshavan M. S. Minshew N. J. (2006). Abnormal brain size effect on the thalamus in autism. Psychiatry Res. 147, 145–151. 10.1016/j.pscychresns.2005.12.009 16945509

[B41] Hardan A. Y. Minshew N. J. Melhem N. M. Srihari S. Jo B. Bansal R.. (2008). An MRI and proton spectroscopy study of the thalamus in children with autism. Psychiatry Res. 163, 97–105. 10.1016/j.pscychresns.2007.12.002 18508243 PMC2467447

[B42] Hunt P. R. Aggleton J. P. (1991). Medial dorsal thalamic lesions and working memory in the rat. Behav. Neural Biol. 55, 227–246. 10.1016/0163-1047(91)80141-z 2059189

[B43] Katz L. C. Shatz C. J. (1996). Synaptic activity and the construction of cortical circuits. Science 274, 1133–1138. 10.1126/science.274.5290.1133 8895456

[B44] Kolb B. Gibb R. (1990). Anatomical correlates of behavioural change after neonatal prefrontal lesions in rats. Prog. Brain Res. 85, 241–255; discussion 255–246. 10.1016/s0079-6123(08)62683-7 2094896

[B45] Kolb B. Gibb R. (2007). Brain plasticity and recovery from early cortical injury. Dev. Psychobiol. 49, 107–118. 10.1002/dev.20199 17299783

[B47] Kolb B. Gibb R. Gorny G. Whishaw I. Q. (1998). Possible regeneration of rat medial frontal cortex following neonatal frontal lesions. Behav. Brain Res. 91, 127–141. 10.1016/s0166-4328(97)00112-5 9578446

[B48] Kolb B. Gibb R. Van der Kooy D. (1994). Neonatal frontal cortical lesions in rats alter cortical structure and connectivity. Brain Res. 645, 85–97. 10.1016/0006-8993(94)91641-1 8062102

[B49] Kolb B. Mychasiuk R. Muhammad A. Li Y. Frost D. O. Gibb R. (2012). Experience and the developing prefrontal cortex. Proc. Natl. Acad. Sci. U S A 109(Suppl. 2), 17186–17193. 10.1073/pnas.1121251109 23045653 PMC3477383

[B46] Kolb B. Nonneman A. J. (1978). Sparing of function in rats with early prefrontal cortex lesions. Brain Res. 151, 135–148. 10.1016/0006-8993(78)90956-3 678999

[B50] Kolb B. Nonneman A. J. Singh R. K. (1974). Double dissociation of spatial impairments and perseveration following selective prefrontal lesions in rats. J. Comp. Physiol. Psychol. 87, 772–780. 10.1037/h0036970 4426996

[B51] Kolb B. Petrie B. Cioe J. (1996). Recovery from early cortical damage in rats, VII. Comparison of the behavioural and anatomical effects of medial prefrontal lesions at different ages of neural maturation. Behav. Brain Res. 79, 1–14. 10.1016/0166-4328(95)00254-5 8883811

[B52] Krettek J. E. Price J. L. (1977). The cortical projections of the mediodorsal nucleus and adjacent thalamic nuclei in the rat. J. Comp. Neurol. 171, 157–191. 10.1002/cne.901710204 64477

[B54] Kuroda M. Murakami K. Kishi K. Price J. L. (1995). Thalamocortical synapses between axons from the mediodorsal thalamic nucleus and pyramidal cells in the prelimbic cortex of the rat. J. Comp. Neurol. 356, 143–151. 10.1002/cne.903560110 7543120

[B55] Kuroda M. Yokofujita J. Murakami K. (1998). An ultrastructural study of the neural circuit between the prefrontal cortex and the mediodorsal nucleus of the thalamus. Prog. Neurobiol. 54, 417–458. 10.1016/s0301-0082(97)00070-1 9522395

[B53] Kuroda M. Yokofujita J. Oda S. Price J. L. (2004). Synaptic relationships between axon terminals from the mediodorsal thalamic nucleus and gamma-aminobutyric acidergic cortical cells in the prelimbic cortex of the rat. J. Comp. Neurol. 477, 220–234. 10.1002/cne.20249 15300791

[B56] Leinekugel X. Tseeb V. Ben-Ari Y. Bregestovski P. (1995). Synaptic GABAA activation induces Ca2+ rise in pyramidal cells and interneurons from rat neonatal hippocampal slices. J. Physiol. 487(Pt. 2), 319–329. 8558466 10.1113/jphysiol.1995.sp020882 PMC1156575

[B57] Leonard C. M. (1969). The prefrontal cortex of the rat. I. Cortical projection of the mediodorsal nucleus. II. Efferent connections. Brain Res. 12, 321–343. 10.1016/0006-8993(69)90003-1 4184997

[B58] Lu B. (2003). BDNF and activity-dependent synaptic modulation. Learn. Mem. 10, 86–98. 10.1101/lm.54603 12663747 PMC5479144

[B59] Lu J. Karadsheh M. Delpire E. (1999). Developmental regulation of the neuronal-specific isoform of K-Cl cotransporter KCC2 in postnatal rat brains. J. Neurobiol. 39, 558–568. 10.1002/(sici)1097-4695(19990615)39:4<558::aid-neu9>3.3.co;2-x 10380077

[B60] Marmolejo N. Paez J. Levitt J. B. Jones L. B. (2012). Early postnatal lesion of the medial dorsal nucleus leads to loss of dendrites and spines in adult prefrontal cortex. Dev. Neurosci. 34, 463–476. 10.1159/000343911 23406908 PMC3884182

[B61] Métin C. Denizot J. P. Ropert N. (2000). Intermediate zone cells express calcium-permeable AMPA receptors and establish close contact with growing axons. J. Neurosci. 20, 696–708. 10632599 10.1523/JNEUROSCI.20-02-00696.2000 PMC6772402

[B62] Minzenberg M. J. Carter C. S. (2012). Developing treatments for impaired cognition in schizophrenia. Trends Cogn. Sci. 16, 35–42. 10.1016/j.tics.2011.11.017 22178120

[B63] Minzenberg M. J. Laird A. R. Thelen S. Carter C. S. Glahn D. C. (2009). Meta-analysis of 41 functional neuroimaging studies of executive function in schizophrenia. Arch. Gen. Psychiatry 66, 811–822. 10.1001/archgenpsychiatry.2009.91 19652121 PMC2888482

[B65] Mitchell A. S. Baxter M. G. Gaffan D. (2007a). Dissociable performance on scene learning and strategy implementation after lesions to magnocellular mediodorsal thalamic nucleus. J. Neurosci. 27, 11888–11895. 10.1523/jneurosci.1835-07.2007 17978029 PMC2241732

[B67] Mitchell A. S. Browning P. G. Baxter M. G. (2007b). Neurotoxic lesions of the medial mediodorsal nucleus of the thalamus disrupt reinforcer devaluation effects in rhesus monkeys. J. Neurosci. 27, 11289–11295. 10.1523/jneurosci.1914-07.2007 17942723 PMC2242856

[B68] Mitchell A. S. Browning P. G. Wilson C. R. Baxter M. G. Gaffan D. (2008). Dissociable roles for cortical and subcortical structures in memory retrieval and acquisition. J. Neurosci. 28, 8387–8396. 10.1523/jneurosci.1924-08.2008 18716197 PMC6671048

[B64] Mitchell A. S. Chakraborty S. (2013). What does the mediodorsal thalamus do? Front. Syst. Neurosci. 7:37. 10.3389/fnsys.2013.00037 23950738 PMC3738868

[B66] Mitchell A. S. Gaffan D. (2008). The magnocellular mediodorsal thalamus is necessary for memory acquisition, but not retrieval. J. Neurosci. 28, 258–263. 10.1523/jneurosci.4922-07.2008 18171943 PMC6671139

[B69] Monyer H. Burnashev N. Laurie D. J. Sakmann B. Seeburg P. H. (1994). Developmental and regional expression in the rat brain and functional properties of four NMDA receptors. Neuron 12, 529–540. 10.1016/0896-6273(94)90210-0 7512349

[B70] Neave N. Sahgal A. Aggleton J. P. (1993). Lack of effect of dorsomedial thalamic lesions on automated tests of spatial memory in the rat. Behav. Brain Res. 55, 39–49. 10.1016/0166-4328(93)90005-b 8329125

[B71] Negyessy L. Goldman-Rakic P. S. (2005). Morphometric characterization of synapses in the primate prefrontal cortex formed by afferents from the mediodorsal thalamic nucleus. Exp. Brain Res. 164, 148–154. 10.1007/s00221-005-2237-6 15776222

[B72] Négyessy L. Hámori J. Bentivoglio M. (1998). Contralateral cortical projection to the mediodorsal thalamic nucleus: origin and synaptic organization in the rat. Neuroscience 84, 741–753. 10.1016/s0306-4522(97)00559-9 9579780

[B73] Nonneman A. J. Corwin J. V. (1981). Differential effects of prefrontal cortex ablation in neonatal, juvenile and young adult rats. J. Comp. Physiol. Psychol. 95, 588–602. 10.1037/h0077800 7053180

[B74] Ongür D. Price J. L. (2000). The organization of networks within the orbital and medial prefrontal cortex of rats, monkeys and humans. Cereb. Cortex 10, 206–219. 10.1093/cercor/10.3.206 10731217

[B75] Owens D. F. Boyce L. H. Davis M. B. Kriegstein A. R. (1996). Excitatory GABA responses in embryonic and neonatal cortical slices demonstrated by gramicidin perforated-patch recordings and calcium imaging. J. Neurosci. 16, 6414–6423. 8815920 10.1523/JNEUROSCI.16-20-06414.1996 PMC6578913

[B76] Owens D. F. Kriegstein A. R. (2002). Is there more to GABA than synaptic inhibition? Nat. Rev. Neurosci. 3, 715–727. 10.1038/nrn919 12209120

[B77] Owens D. F. Liu X. Kriegstein A. R. (1999). Changing properties of GABA(A) receptor-mediated signaling during early neocortical development. J. Neurophysiol. 82, 570–583. 10444657 10.1152/jn.1999.82.2.570

[B78] Pakkenberg B. (1990). Pronounced reduction of total neuron number in mediodorsal thalamic nucleus and nucleus accumbens in schizophrenics. Arch. Gen. Psychiatry 47, 1023–1028. 10.1001/archpsyc.1990.01810230039007 2241504

[B79] Parnaudeau S. O’neill P.-K. Bolkan S. S. Ward R. D. Abbas A. I. Roth B. L.. (2013). Inhibition of mediodorsal thalamus disrupts thalamofrontal connectivity and cognition. Neuron 77, 1151–1162. 10.1016/j.neuron.2013.01.038 23522049 PMC3629822

[B80] Pirot S. Jay T. M. Glowinski J. Thierry A. M. (1994). Anatomical and electrophysiological evidence for an excitatory amino acid pathway from the thalamic mediodorsal nucleus to the prefrontal cortex in the rat. Eur. J. Neurosci. 6, 1225–1234. 10.1111/j.1460-9568.1994.tb00621.x 7524967

[B81] Popken G. J. Bunney W. E. Jr. Potkin S. G. Jones E. G. (2000). Subnucleus-specific loss of neurons in medial thalamus of schizophrenics. Proc. Natl. Acad. Sci. U S A 97, 9276–9280. 10.1073/pnas.150243397 10908653 PMC16858

[B82] Quinlan E. M. Olstein D. H. Bear M. F. (1999). Bidirectional, experience-dependent regulation of N-methyl-D-aspartate receptor subunit composition in the rat visual cortex during postnatal development. Proc. Natl. Acad. Sci. U S A 96, 12876–12880. 10.1073/pnas.96.22.12876 10536016 PMC23143

[B83] Righi G. Tierney A. L. Tager-Flusberg H. Nelson C. A. (2014). Functional connectivity in the first year of life in infants at risk for autism spectrum disorder: an EEG study. PLoS One 9:e105176. 10.1371/journal.pone.0105176 25140874 PMC4139321

[B84] Rios O. Villalobos J. (2004). Postnatal development of the afferent projections from the dorsomedial thalamic nucleus to the frontal cortex in mice. Brain Res. Dev. Brain Res. 150, 47–50. 10.1016/j.devbrainres.2004.02.005 15126037

[B85] Rivera C. Voipio J. Payne J. A. Ruusuvuori E. Lahtinen H. Lamsa K.. (1999). The K+/Cl− co-transporter KCC2 renders GABA hyperpolarizing during neuronal maturation. Nature 397, 251–255. 10.1038/16697 9930699

[B86] Rose J. E. Woolsey C. N. (1948). The orbitofrontal cortex and its connections with the mediodorsal nucleus in rabbit, sheep and cat. Res. Publ. Assoc. Res. Nerv. Ment. Dis. 27(Vol. 1), 210–232. 18106857

[B87] Rotaru D. C. Barrionuevo G. Sesack S. R. (2005). Mediodorsal thalamic afferents to layer III of the rat prefrontal cortex: synaptic relationships to subclasses of interneurons. J. Comp. Neurol. 490, 220–238. 10.1002/cne.20661 16082676

[B88] Schlaepfer T. E. Harris G. J. Tien A. Y. Peng L. W. Lee S. Federman E. B.. (1994). Decreased regional cortical gray matter volume in schizophrenia. Am. J. Psychiatry 151, 842–848. 8184992 10.1176/ajp.151.6.842

[B89] Sheng M. Cummings J. Roldan L. A. Jan Y. N. Jan L. Y. (1994). Changing subunit composition of heteromeric NMDA receptors during development of rat cortex. Nature 368, 144–147. 10.1038/368144a0 8139656

[B90] Sherman S. M. (2014). The function of metabotropic glutamate receptors in thalamus and cortex. Neuroscientist 20, 136–149. 10.1177/1073858413478490 23459618 PMC4747429

[B91] Snyder M. A. Gao W. J. (2013). NMDA hypofunction as a convergence point for progression and symptoms of schizophrenia. Front. Cell. Neurosci. 7:31. 10.3389/fncel.2013.00031 23543703 PMC3608949

[B92] Staley K. J. Soldo B. L. Proctor W. R. (1995). Ionic mechanisms of neuronal excitation by inhibitory GABAA receptors. Science 269, 977–981. 10.1126/science.7638623 7638623

[B93] Stokes K. A. Best P. J. (1988). Mediodorsal thalamic lesions impair radial maze performance in the rat. Behav. Neurosci. 102, 294–300. 10.1037//0735-7044.102.2.294 3365324

[B94] Tang G. Gudsnuk K. Kuo S. H. Cotrina M. L. Rosoklija G. Sosunov A.. (2014). Loss of mTOR-dependent macroautophagy causes autistic-like synaptic pruning deficits. Neuron 83, 1131–1143. 10.1016/j.neuron.2014.07.040 25155956 PMC4159743

[B95] Tanibuchi I. Goldman-Rakic P. S. (2003). Dissociation of spatial-, object- and sound-coding neurons in the mediodorsal nucleus of the primate thalamus. J. Neurophysiol. 89, 1067–1077. 10.1152/jn.00207.2002 12574481

[B96] Tsatsanis K. D. Rourke B. P. Klin A. Volkmar F. R. Cicchetti D. Schultz R. T. (2003). Reduced thalamic volume in high-functioning individuals with autism. Biol. Psychiatry 53, 121–129. 10.1016/s0006-3223(02)01530-5 12547467

[B97] Uylings H. B. Groenewegen H. J. Kolb B. (2003). Do rats have a prefrontal cortex? Behav. Brain Res. 146, 3–17. 10.1016/j.bbr.2003.09.028 14643455

[B98] Van Eden C. G. (1986). Development of connections between the mediodorsal nucleus of the thalamus and the prefrontal cortex in the rat. J. Comp. Neurol. 244, 349–359. 10.1002/cne.902440307 3958232

[B100] van Eden C. G. Kros J. M. Uylings H. B. (1990). The development of the rat prefrontal cortex. Its size and development of connections with thalamus, spinal cord and other cortical areas. Prog. Brain Res. 85, 169–183. 10.1016/s0079-6123(08)62680-1 2094893

[B99] Van Eden C. G. Uylings H. B. (1985). Postnatal volumetric development of the prefrontal cortex in the rat. J. Comp. Neurol. 241, 268–274. 10.1002/cne.902410303 4086657

[B101] van Eden C. G. van Hest A. van Haaren F. Uylings H. B. (1994). Effects of neonatal mediodorsal thalamic lesions on structure and function of the rat prefrontal cortex. Brain Res. Dev. Brain Res. 80, 26–34. 10.1016/0165-3806(94)90086-8 7955351

[B102] Vicedomini J. P. Corwin J. V. Nonneman A. J. (1982). Behavioral effects of lesions to the caudate nucleus or mediodorsal thalamus in neonatal, juvenile and adult rats. Physiol. Psychol. 10, 246–250 10.3758/bf03332944

[B103] Wang H. X. Stradtman G. G. R. Wang X. J. Gao W. J. (2008). A specialized NMDA receptor function in layer 5 recurrent microcircuitry of the adult rat prefrontal cortex. Proc. Nat. Acad. Sci. U S A 105, 16791–16796. 10.1073/pnas.0804318105 18922773 PMC2575498

[B104] Wang M. Yang Y. Wang C.-J. Gamo N. J. Jin L. E. Mazer J. A.. (2013). NMDA receptors subserve persistent neuronal firing during working memory in dorsolateral prefrontal cortex. Neuron 77, 736–749. 10.1016/j.neuron.2012.12.032 23439125 PMC3584418

[B105] Watanabe Y. Funahashi S. (2012). Thalamic mediodorsal nucleus and working memory. Neurosci. Biobehav. Rev. 36, 134–142. 10.1016/j.neubiorev.2011.05.003 21605592

[B106] Winocur G. (1985). The hippocampus and thalamus: their roles in short- and long-term memory and the effects of interference. Behav. Brain Res. 16, 135–152. 10.1016/0166-4328(85)90088-9 4041213

[B107] Wise S. P. Jones E. G. (1978). Developmental studies of thalamocortical and commissural connections in the rat somatic sensory cortex. J. Comp. Neurol. 178, 187–208. 10.1002/cne.901780202 627623

[B108] Wolff J. R. Joó F. Dames W. (1978). Plasticity in dendrites shown by continuous GABA administration in superior cervical ganglion of adult rat. Nature 274, 72–74. 10.1038/274072a0 661998

[B109] Yaklovev P. I. Lecours A. (1967). “The myelogenetic cycles of regional maturation of the brain,” in Regional Development of the Brain in Early Life, ed Minkowski A. (Oxford: Blackwell Science), 3–70.

[B110] Young K. A. Manaye K. F. Liang C. Hicks P. B. German D. C. (2000). Reduced number of mediodorsal and anterior thalamic neurons in schizophrenia. Biol. Psychiatry 47, 944–953. 10.1016/s0006-3223(00)00826-x 10838062

[B111] Zipursky R. B. Lim K. O. Sullivan E. V. Brown B. W. Pfefferbaum A. (1992). Widespread cerebral gray matter volume deficits in schizophrenia. Arch. Gen. Psychiatry 49, 195–205. 10.1001/archpsyc.1992.01820030027004 1567274

